# Shared care in surgery: Practical considerations for surgical leaders

**DOI:** 10.1177/0840470420952485

**Published:** 2020-09-01

**Authors:** Morgann Reid, Alex Lee, David R. Urbach, Craig Kuziemsky, Morad Hameed, Husein Moloo, Fady Balaa

**Affiliations:** 1 56004 Telfer School of Management, University of Ottawa, Ottawa, Ontario, Canada.; 2 12365 Faculty of Medicine, University of Ottawa, Ottawa, Ontario, Canada.; 3 7985 Division of General Surgery, Department of Surgery, University of Toronto, Toronto, Ontario, Canada.; 4 Office of Research Services and School of Business, 3151 MacEwan University, Edmonton, Alberta, Canada.; 5 Division of General Surgery, 199005 Department of Surgery, University of British Columbia, Vancouver, British Columbia, Canada.; 6 12365 Division of General Surgery, Department of Surgery, University of Ottawa, Ottawa, Ontario, Canada.

## Abstract

The recent COVID-19 pandemic has highlighted limitations in current healthcare systems and needed strategies to increase surgical access. This article presents a team-based integration model that embraces intra-disciplinary collaboration in shared clinical care, professional development, and administrative processes to address this surge in demand for surgical care. Implementing this model will require communicating the rationale for and benefits of shared care, while shifting patient trust to a team of providers. For the individual surgeon, advantages of clinical integration through shared care include decreased burnout and professional isolation, and more efficient transitions into and out of practice. Advantages to the system include greater surgeon availability, streamlined disease site wait lists, and promotion of system efficiency through a centralized distribution of clinical resources. We present a framework to stimulate national dialogue around shared care that will ultimately help overcome system bottlenecks for surgical patients and provide support for health professionals.

## Introduction

Surgical leaders will be searching for strategies to navigate the post COVID-19 surge in demand for surgical access.^1^ A commentary in the *Canadian Medical Association Journal* recently highlighted team-based Single-Entry Models (SEMs) as essential in managing this demand.^2^ SEMs, rooted in queuing theory, allow patients to see the next-available surgeon from a group of physicians with the same practice profile.^3^ Although SEMs are garnishing increased support, leveraging the full potential of team-based surgical care requires a framework that considers the many bottlenecks beyond the patient’s entry into the healthcare system.

Shifting towards a team-based shared care approach will be challenging for surgical programs that function in the traditional solo surgeon model. Such a change will require a significant investment from leaders to understand, design, and disseminate a framework for shared care in their respective programs.^4^ As surgical leaders now face heavy administrative burdens related to the COVID-19 pandemic, it will be difficult to enact such a major change in surgical care. This creates space for a national dialogue to discuss ideas and strategies around shared care models in surgery. In this paper, we share a successful framework that can be used as a toolkit for achieving team-based shared care which has been active for 6 years in an academic general surgery division.

While much emphasis has been placed on describing and implementing inter-disciplinary and multidisciplinary care,^5–9^ there has been limited progress in defining the mechanics of *intra*-disciplinary collaboration to achieve team-based shared care.^10^ Intra-disciplinary collaboration occurs when surgeons within the same discipline are embedded in each other’s practice to enhance knowledge transfer, develop skills, and deliver better patient care. Intra-disciplinary collaboration is the crux of team-based shared care; the capabilities of the collective are harnessed for shared clinical care, professional development, and to overcome bottlenecks in administrative processes related to resource allocation and utilization. Team-based shared care could prove useful in navigating surges in surgical demand related to the COVID-19 pandemic and could prove to be a safer model of care for patients in the long term.^5^

## Implementation process

### Step 1: Communicating the rationale of team-based shared care

The process of transitioning towards a team-based model of shared care can bring significant disruption to the traditional solo surgeon practice workflow that exists in most surgical programs.^11^ Understandably, the most consequential concern typically revolves around the fear of degrading the element of trust that is central to the doctor-patient relationship.^12^ Surgeons, other healthcare providers, and administrative staff need to accept the rationale for team-based shared care, believe in the benefits of clinical integration, and be assured that trust can feasibly be shifted from trust in an individual provider to trust in a team of providers. This shift is exemplified by the airline industry which has secured passenger trust to the point where passengers are indifferent to who is piloting their flight.^13^ All pilots are “equivalent actors” in skills and training and are interchangeable. As a result, passenger trust is built with the system rather than individuals.^13^ Similarly, acute care surgery models, emergency obstetrics and gynecology care models, and bariatric surgery networks rely on interchangeable providers and highlight the receptibility and feasibility of broad adoption of integrated models in surgery.^14–16^

The rationale and process for team-based shared care in surgery has to be communicated transparently and described in terms of benefits to patients, providers, and the system as a whole.^17^ At our institution, this was conveyed using a two-pronged approach through formal division-wide administrative meetings, and through informal discussion between leadership and individual providers who were able to provide their perspective and feedback on a team-based shared care model. Ultimately, the intent of team-based shared care is to build system resiliency and necessary redundancy. Multiple surgeons sharing the care of a patient decreases the risk of professional isolation and burnout.^18–21^ It also creates a natural mechanism for coaching environments and overlapping transitions into practice for junior surgeons and out of practice for retiring surgeons.^22,23^ Through team-based shared care, the healthcare system benefits from overall increased surgeon availability (eg, continual coverage for a surgeon during vacation, parental, academic leave, etc), efficient resource utilization, patients single-entry into care, and minimization of heterogeneous wait lists.^24–26^ In an effort to enhance efficiency and better address increasing demands for surgical access, a team-based model can leverage the centralized distribution of clinical resources (including elective operating rooms, endoscopy units, and outpatient clinical access) to clinical programs and not to individual surgeons.^24–26^

### Step 2: Grouping disease sites into SubSpecialty Units

A team-based model for shared care naturally lends itself to grouping disease sites into “SubSpeciality Units” (SSU). Table 1 provides an example of SSUs that cover the spectrum of care in academic divisions of general surgery. This positions patients well for a single-entry into the healthcare system through *disease site wait lists* as opposed to *surgeon specific wait lists*. While this restructuring may represent challenging transitions for surgeons who are accustomed to very broad practice profiles, it is an essential second step in developing a clear reporting structure around clinical service delivery to drive an agenda of team-based surgical care.

**Table 1.** Subspecialty units in an academic division of general surgery

1	Core surgery (includes surgeons with a practice profile focused on acute care surgery, trauma, and abdominal wall reconstruction)
2	Colorectal surgery
3	General surgical oncology
4	Foregut and minimally invasive surgery
5	Breast surgical oncology
6	Hepatobiliary and pancreatic surgery

Each SSU represents a team-based clinical program, with an assigned “section head” who serves as the administrative lead responsible for clinical oversight and coordination. Relevant tasks for the section head could include appropriate resource allocation, leading program-specific committees, leading the implementation of clinical pathways, liaising with hospital or cross-organizational administration, and quality assurance and improvement. Higher-order leadership can leverage this disease site organizational approach to structure team-based shared care, and to serve in the development of robust clinically integrated programs, moving the focus away from individual surgeon practices.

### Step 3: Implementation of an SSU to facilitate shared care

In a feasible model of team-based shared care, the day-to-day workflow should embed surgeons in each other’s practices such that collaboration is the natural route. This organically offers support for the provider and improves workflow efficiency for the system.^20,23,26^ This can be achieved by implementing certain mechanisms that facilitate team-based shared care within an SSU (Table 2). These mechanisms can be bundled into four themes: administrative operation, resource utilization, shared clinical care, and shared teaching.

**Table 2.** Toolkit to help guide the practical implementation of a subspecialty unit (SSU) within a surgical program

Theme	Mechanism
Administrative operation within an SSU	1. Share physical office space. 2. Share and manage administrative assistant pool with shared phone coverage. 3. Share and manage a service schedule including reasonable coverage during holidays. 4. Manage a single-entry model (ie, a single queue) for new referral, to provide sufficient coverage for the region.
Resource utilization within an SSU	5. Manage blocked distribution of operating room access through the SSU. 6. Manage blocked distribution of endoscopy access through the SSU. 7. Manage blocked distribution of outpatient clinic access through the SSU. 8. Arrange a single-entry (ie, a single queue) for operating room booking.
Shared clinical care within an SSU	9. Arrange for a shared inpatient service coverage. “Surgeon of the week” model for managing the inpatient service. 10. Participate in a simultaneous outpatient clinic with another one or more surgeons who have a relatively similar practice profile. 11. Participate in a shared specialized treatment (eg, endoscopy) or operating room consent process with one or more surgeons who have a relatively similar practice profile. Receive patient consent for multiple surgeons, and treatment by the next available surgeon. 12. Regularly participate in “co-surgery” where two surgeons with the same specialty operate together on double scrubbed cases. 13. Share adoption of clinical pathway and care maps.
Shared teaching within an SSU	14. Share participation in clinical teaching. 15. Arrange regular subspecialty rounds.

Through a shared administrative operation within an SSU, outpatients have improved access to the practice through an SEM and shared phone coverage. In addition, a shared administrative operation can enable a system of smart scheduling that can best match the centrally distributed resources with surgeon availability, which is often complicated by non-clinical responsibilities, including administrative, teaching, and other academic and scholarly duties.^27^ The mechanisms under shared clinical care allow for multi-surgeon input into care both inside (eg, surgeons performing operations with partners through “co-surgery”) and outside the operating room.^28^ This includes collective adoption of clinical pathways and process maps that facilitate standardized care and grant opportunities for quality improvement. Inpatient service coverage is shared under a “surgeon of the week” model where a different surgeon in the team is responsible for triaging new referrals and supervising care for admitted patients each week. Moreover, by sharing in teaching responsibilities, surgeons can model collaboration to trainees, and in-turn, benefit from an environment loaded with frequent peer-peer learning.^22,23^

Implementing all 15 mechanisms for all members of a surgical program might not be realistic in the short term, however with repeated messaging and effort from leadership, the toolkit can be gradually adopted within a few years. In a recent internal audit five years after initiation of an SSU framework in an academic division (University of Ottawa Division of General Surgery), all units have made significant progress toward implementation of SSUs and team-based collaboration. During this 5-year transition, the division experienced a favourable growth of faculty size by 32% (10 new surgeons), a 7% increase in surgical volumes, and a 35% increase in outpatient clinic visits. The division also advanced academic productivity as the number of surgeons and residents leading or collaborating on scholarly publications grew from 13 to 75, over the same time period. In response to the COVID-19 pandemic, adoption of these mechanisms can streamline preparation to resume elective surgeries but also help to meet the demands in increasing surgical capacity.^29^

## Conclusion

Surgeons and the systems they work within are strategizing for ways to accommodate the drastic spike in demand for surgical access related to the pandemic. This crisis and the reflection on the limitations in the current system can bring an opportunity to exchange traditional isolating single surgeon models for more integrated team-based shared surgical care. To achieve this, the system architecture must be consistent with its goals. Such a model of care, built on intra-disciplinary collaboration, is applicable and adaptable in other disciplines beyond those in the surgical space that are also challenged with increasing demands and can benefit from the collaboration of multiple providers who share similar practice profiles. Future work to further validate the SSU toolkit with key stakeholders, assess provider and patient experience, and measure system efficiency will improve adoption and further refine functioning of this team-based shared care model.

The elements presented in this toolkit are all based on a premise of a system with increased intra-disciplinary collaboration which facilitates a shift from surgeon specific wait lists to disease site wait lists and addresses the sequential provider-related bottlenecks that can exist in solo surgical practice. This can be achieved while retaining the element of trust between the patient and the team, and improving the provider experience for the individual surgeon.
